# U.S. census unit population exposures to ambient air pollutants

**DOI:** 10.1186/1476-072X-11-3

**Published:** 2012-01-12

**Authors:** Yongping Hao, Helen Flowers, Michele M Monti, Judith R Qualters

**Affiliations:** 1 National Center for Environmental Health, Centers for Disease Control and Prevention, Atlanta, Georgia

**Keywords:** Census geographic unit, concentration, population exposure, ambient air pollutants, PM_2.5_, ozone

## Abstract

**Background:**

Progress has been made recently in estimating ambient PM_2.5 _(particulate matter with aerodynamic diameter < 2.5 μm) and ozone concentrations using various data sources and advanced modeling techniques, which resulted in gridded surfaces. However, epidemiologic and health impact studies often require population exposures to ambient air pollutants to be presented at an appropriate census geographic unit (CGU), where health data are usually available to maintain confidentiality of individual health data. We aim to generate estimates of population exposures to ambient PM_2.5 _and ozone for U.S. CGUs.

**Methods:**

We converted 2001-2006 gridded data, generated by the U.S. Environmental Protection Agency (EPA) for CDC's (Centers for Disease Control and Prevention) Environmental Public Health Tracking Network (EPHTN), to census block group (BG) based on spatial proximities between BG and its four nearest grids. We used a bottom-up (fine to coarse) strategy to generate population exposure estimates for larger CGUs by aggregating BG estimates weighted by population distribution.

**Results:**

The BG daily estimates were comparable to monitoring data. On average, the estimates deviated by 2 μg/m^3 ^(for PM_2.5_) and 3 ppb (for ozone) from their corresponding observed values. Population exposures to ambient PM_2.5 _and ozone varied greatly across the U.S. In 2006, estimates for daily potential population exposure to ambient PM_2.5 _in west coast states, the northwest and a few areas in the east and estimates for daily potential population exposure to ambient ozone in most of California and a few areas in the east/southeast exceeded the National Ambient Air Quality Standards (NAAQS) for at least 7 days.

**Conclusions:**

These estimates may be useful in assessing health impacts through linkage studies and in communicating with the public and policy makers for potential intervention.

## Background

Air pollution monitoring data has customarily been compiled and maintained by the EPA and/or state and local agencies. These data have been used in several studies that found ambient air pollutants associated with mortality [1-4] and morbidity [5-9]. However, air monitoring sites are typically sparsely located in very limited geographic areas - only 20% of U.S. counties have at least one monitoring station for PM_2.5 _- and the temporal resolution and type of pollutants measured vary by station (e.g., PM_2.5 _data is only available about every 3-6 days). Thus, studies based on monitoring data were usually limited to high population density areas such as cities or urban/suburban centers, where most monitoring stations are located.

To expand geographic coverage and increase temporal resolution of air pollution data, several studies have recently estimated ambient air pollution concentrations using various data sources and advanced modeling techniques [10-13]. Thus, areas with very sparse or no monitoring data now have gridded data with a variety of spatial (e.g., 4 km, 36 km) and temporal (e.g., hourly, daily) resolutions. However, these data have not been widely accepted by health researchers partly because studies of possible effects of ambient air pollutants on human health often require population exposures to ambient air pollutants to be presented at certain census geographic levels (e.g., census tract, county), where health data are usually available to maintain confidentiality of individual health data [14,15]. Other socioeconomic and demographic data are also routinely collected at such geographic resolutions [16].

Ideally, concentration should be presented at the finest CGU possible, at which air pollution concentration may approximate the potential population exposure to a certain kind of ambient air pollutant, whereas actual population exposure may be close to zero in certain places where few people live (e.g., mountains), no matter how high the concentration of pollutants. From a public health perspective, it is the exposure that makes people sick. The goal of this study is, therefore, to estimate CGU population exposures to ambient PM_2.5 _and ozone. Two major steps are taken to achieve this goal: 1) estimate BG daily ambient PM_2.5 _and ozone concentrations from the gridded data and conduct data comparisons against ground-based monitoring values; and 2) aggregate BG concentrations to generate population exposure estimates for larger CGUs using BG population as a weighting factor. We choose BG (instead of census block) as the basic unit because BG is the lowest CGU where population data are available on an annual basis. BGs generally contain between 600 and 3,000 people, with an optimum population size of 1,500 [17].

## Materials and methods

### Data source: gridded PM_2.5 _and ozone concentrations

Gridded PM_2.5 _(μg/m^3^) and ozone (ppb) concentrations were obtained using a hierarchical Bayesian model developed by the EPA for CDC's EPHTN [12], which provide 24-hour maximum PM_2.5 _and 8-hour maximum ozone concentrations on a daily basis (2001-2006). The model uses source-based Community Multiscale Air Quality (CMAQ) model outputs and monitoring data. It accounts for spatial and temporal dependencies of air pollutants through a hierarchical Bayesian approach. The spatial resolution of data was inherited from CMAQ modeling outputs. CMAQ considered information about emission inventories, meteorological information, and land use. The detailed information about CMAQ and monitoring data can be obtained from http://epa.gov/asmdnerl/CMAQ[18] and http://airnow.gov[19], respectively. The model resulted in two sets of gridded data: 36 km grid-cells for the contiguous U.S. and 12 km grid-cells for an eastern portion of the U.S., which includes the Northeast census region and the South Atlantic and East South Central divisions of the South census region (excluding part of south Florida) and part of Arkansas and Louisiana; portions of the Midwest census region, which includes the entire East North Central division and part of Minnesota, Iowa and Missouri (http://www.census.gov/geo/www/us_regdiv.pdf) [20]. The gridded data fill "holes" in both time (when data are missing on certain days) and space (locations where data are not available). Information on CDC's ongoing EPHTN has been described elsewhere [21,22] and is also available from http://www.cdc.gov/ephtracking[23].

### Estimating BG PM_2.5 _and ozone concentrations

We used a distance-weighting method to estimate BG daily PM_2.5 _and ozone concentrations for all U.S. BGs based on 36 km-gridded data (12 km-gridded data for an eastern portion of the U.S.). Empirical studies, which compared different methods of areal interpolation, suggested that distance-weighting was an appropriate method in calculating population exposure estimates [10,24]; distance-weighting relaxes the homogeneity assumption associated with area-weighting method and overcomes bias introduced by the equal contribution assumption associated with internal or nearest neighboring method.

**Figure 1:**
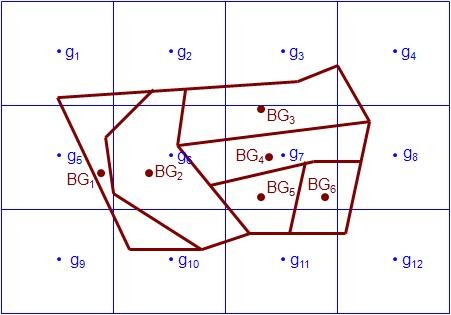
**The demonstration of spatial relationships between BGs and grids**. The four nearest neighboring grids (g) for BGs 1-6: BG1: g5, g6, g9, and g10; BG2: g5, g6, g9, and g10; BG3: g2, g3, g6, and g7; BG4: g3, g6, g7, and g11; BG5: g6, g7, g10, and g11; BG6: g7, g8, g11, and g12.

To make the calculations, the following steps were taken. First, the distance between the centroid of each BG and the corresponding four nearest grids (centroids) were calculated using the newly developed GEODIST function available in SAS software, version 9.2 [25]. The GEODIST function uses the Vincenty distance formula to compute the geodetic distance between any two arbitrary latitude and longitude coordinates in terms of degrees or in radians [26]. The Vincenty-based computation used by the GEODIST function is more accurate than the most commonly used method of the Haversine distance formula [27]. In this study, we used degrees in the GEODIST function. Each BG is associated with the nearest four neighboring grids for both 36 km and 12 km data. Figure 1 demonstrates some possible spatial relationships between BGs and grids. In urban areas, many BGs are located within a 36 km or 12 km grid.

Second, BG daily PM_2.5 _and ozone concentrations were calculated using the inverse of the squared distance as a weighting factor. The inverse of the squared distance is the most commonly used format of distance-weighting, which gives higher weight to closer observations [10,24]. The weight *w_i _*for one of the four neighboring grids nearest to a BG centroid was calculated as

$$w_{i}=\left(1∕d_{i}^{2}\right)∕\left(\overset{4}{\underset{i=1}{\sum }}1∕d_{i}^{2}\right)$$

where *d_i _*is the distance between a grid centroid and a BG centroid. Two separate estimates were derived for the U.S. (from 36km gridded data) and an eastern portion of the U.S. (from 12km gridded data). Daily PM_2.5 _or ozone concentration *μ_i _*for a BG was estimated as

$$\mu_{i}=\overset{4}{\underset{i=1}{\sum }}w_{i}*P_{i}$$

where *P_i _*is the corresponding neighboring grid's concentration measure for PM_2.5 _or ozone. The variance ${\delta_{i}}^{2}$ associated with a BG was estimated as

$${\delta_{i}}^{2}=\overset{4}{\underset{i=1}{\sum }}w_{i}^{2}\sigma_{i}^{2}$$

where *δ_i _*is the standard error associated with BG estimate *μ_i_*; and σ*_i _*is the standard error associated with the original grid's concentration measure P*_i_*.

Third, the derived 2001-2006 BG daily estimates for PM_2.5 _and ozone were compared with monitoring data observed at ground stations within each BG boundary. The comparison was restricted to the area equivalent to 12 km grid-cell coverage (i.e, an eastern portion of the U.S.) for simplicity of having estimates from both 36 km- and 12 km-gridded data. The number of monitoring sites in each BG ranges from 0 to 2. The comparison was conducted for those BGs containing 1 or 2 monitoring sites. The majority of BGs contained only one monitoring site (e.g., 1055 BGs contains one versus 26 BGs contains two PM_2.5 _monitoring sites). We calculated two statistics for data comparison: mean absolute deviation (MAD), an intuitive measure of absolute fit; and correlation coefficient (*R*), a measure of relative fit. MAD measures the average absolute deviations of the estimates from their corresponding observed data [28]. In time series analysis, MAD measures the average absolute deviation of observations from their forecasts.

### Estimating daily population exposures to ambient PM_2.5 _and ozone for larger CGUs

Unlike concentration, exposure comes with population; no population, no exposure. Population exposure could substantially differ from concentration itself depending on population distribution within a CGU. Therefore, when estimating population exposure for a CGU, population distribution needs to be factored in. Using BG as a base, we aggregated BG concentration to generate larger CGU population exposure measure by taking into account the population location of BG. This method has been used in generating population exposure to ambient air pollutant for larger graphic areas based on estimates at a fine spatial resolution [8,29,30]. The daily population exposure to ambient air pollutant (*PE*) at a larger CGU, such as census tract, was estimated as

$$PE=\overset{n}{\underset{i=1}{\sum }}\left(\frac{pop_{i}}{\overset{4}{\underset{i=1}{\sum }}pop_{i}}\right)*\mu_{i}$$

where *n *is the number of BGs within a CGU and *pop_i _*is the BG population; similarly the standard error (*ψ*) associated with the population exposure to ambient air pollutant (*PE*) at a CGU was estimated as

$$\psi =\sqrt{\overset{n}{\underset{i=1}{\sum }}{\left(\frac{pop_{i}}{\overset{n}{\underset{i=1}{\sum }}pop_{i}}\right)}^{2}*\delta_{i}^{2}}$$

where *δ_i _*is the standard error associated with BG estimate, *μ_i_*. In this study, we generated daily population exposure estimates (*PEs *and their standard errors *ψs*) (2001-2006) for census tract, county, state and the U.S. accordingly by aggregating BG daily estimates weighted by BG population distribution across corresponding larger CGUs.

We mapped the 98^th ^percentiles of 2006 daily population exposures to ambient PM_2.5 _and ozone for census tract and county to demonstrate geographic variation in population exposures to ambient PM_2.5 _and ozone and highlight where severe population exposures to these two ambient pollutants could potentially occurs. The 98^th ^percentile of 2006 daily population exposure estimate shows the seventh-highest daily population exposure (i.e., 2% of 365 days equals to ~7 days) that the population in a CGU has experienced in that year. We grouped population exposure to ambient air pollutants into five categories. The cut point for the second highest category is adjusted to match the NAAQS (daily 24-hour standard of 35 μg/m^3 ^for PM_2.5 _and daily 8-hour standard of 75 ppb for ozone) [31] and the highest one and the lowest three categories are set at equal lengths of 10 units above or below the standards (e.g., cut points for the highest ones are set as 45 for PM_2.5 _and 85 for ozone). ArcGIS software was used in mapping [32]. Similarly, we mapped the 90^th ^percentiles of 2006 daily population exposures to ambient PM_2.5 _and ozone for census tract and county with five manually grouped categories (the highest category is set to match the NAAQS) to allow a spatial pattern to emerge. The 90^th ^percentile of 2006 daily population exposure estimate corresponds to the thirty fifth-highest daily population exposure (i.e., 10% of 365 days equals to ~35 days) that the population in a CGU has experienced in that year.

We further calculated the number of days when PM_2.5 _or ozone concentration exceeded the NAAQS for each BG. The populations at risk for larger CGUs were calculated by aggregating BG populations with exposure to ambient PM_2.5 _or ozone exceeding the NAAQS.

## Results

### Data comparison: BG estimates against ground-based monitoring data

The mean of BG 24-hour maximum PM_2.5 _and 8-hour maximum ozone estimate for an entire year across the U.S. is 13 μg/m^3 ^and 44 ppb, respectively. We presented two comparison statistics between BG daily estimates and ground-based monitoring data in an eastern portion of the U.S., by year, in Table 1 along with number of monitoring sites (M) and number of observations (N). On average, the estimates deviated by 2 μg/m^3 ^(for PM_2.5_) and 3 ppb (for ozone) from their corresponding observed values. The MADs of BG estimates based on the 36 km-gridded data were similar to those resulted from 12 km-gridded data. Although the former was slightly smaller than the later, the difference between the two was very small. Similar patterns were observed for correlation coefficient *R *(Table 1). In addition to MAD, we compared other distributional statistics of absolute deviation between predicted and observed (i.e., minimum, median, maximum, and the 5^th^, 10^th^, 90^th^, and 95^th ^percentiles); and we observed little discrepancy by season, by year, or by urban vs. rural status between the two (Additional file 1).

**Table 1:** Comparison statistics between BG daily estimates and ground-based monitoring data in an eastern portion of the U.S., by year

Concentration (unit)	Year	M	N	MAD (12 km grid)	MAD (36 km grid)	R (12 km grid)	R (36 km grid)
**PM_2.5 _**(μg/m^3^)	2001	813	113401	2.23	1.69	0.89	0.93
**PM_2.5 _**(μg/m^3^)	2002	899	127779	2.31	1.55	0.88	0.95
**PM_2.5 _**(μg/m^3^)	2003	894	117879	2.36	1.56	0.88	0.94
**PM_2.5 _**(μg/m^3^)	2004	833	116326	2.25	1.47	0.89	0.95
**PM_2.5 _**(μg/m^3^)	2005	860	111552	2.41	1.58	0.90	0.95
**PM_2.5 _**(μg/m^3^)	2006	807	105158	2.18	1.42	0.89	0.95
**Ozone **(ppb)	2001	790	195035	4.53	3.22	0.94	0.97
**Ozone **(ppb)	2002	856	223175	3.83	3.23	0.96	0.97
**Ozone **(ppb)	2003	873	226947	3.69	3.07	0.96	0.97
**Ozone **(ppb)	2004	896	232081	3.51	2.98	0.95	0.96
**Ozone **(ppb)	2005	883	230270	3.75	3.09	0.96	0.97
**Ozone **(ppb)	2006	887	227576	3.63	3.01	0.95	0.96

M: The number of monitoring sites; N: The number of records;

MAD: Mean absolute deviation; R: Correlation coefficient

### Estimating CGU population exposures to ambient PM_2.5 _and ozone

**Figure 2:**
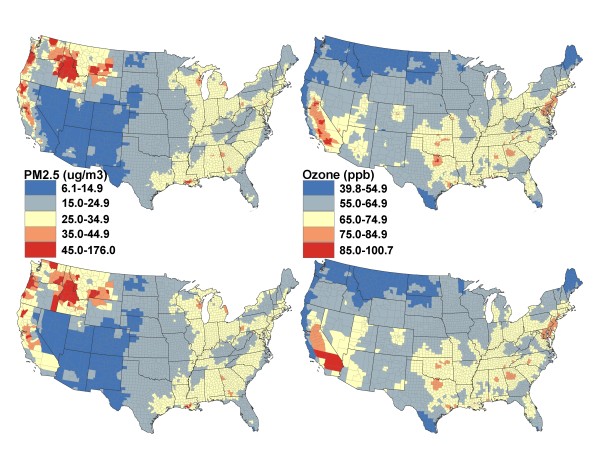
**The 98^th ^percentiles of estimated daily potential population exposures to ambient PM_2.5 _and ozone in 2006**. [The upper two panels correspond to Census tract and the lower two panels correspond to County].

**Figure 3:**
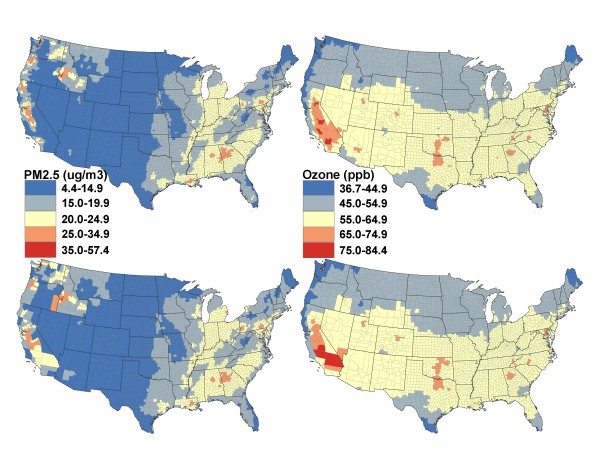
**The 90^th ^percentiles of estimated daily potential population exposures to ambient PM_2.5 _and ozone in 2006**. [The upper two panels correspond to Census tract and the lower two panels correspond to County].

Figure 2 shows the 98^th ^percentiles of 2006 daily potential population exposure to ambient PM_2.5 _and ozone for census tracts and counties. The patterns showed by census tract (upper two panels) were similar to those captured at county level (lower two panels) for most of the U.S., especially the eastern U.S., for both PM_2.5 _and ozone. However, there were visible differences between patterns revealed at census tract level and at county level for west coast areas (e.g., California) for both PM_2.5 _and ozone (Figure 2). Daily potential population exposure to ambient PM_2.5 _was the lowest in the southwest (< 15 μg/m^3^) except California; such exposure increased when moving east; whereas the highest daily potential population exposure to ambient PM_2.5 _occurred in west coast and northwest areas, which exceeded the NAAQS of 35 μg/m^3 ^for 7 days. There were some obvious locations in the east where the daily potential population exposure to ambient PM_2.5 _was above the standard for 7 days (Figure 2, left two panels). Daily potential population exposure to ambient ozone was the lowest in the northwest and increased when moving south and southeast (or southwest). Daily potential population exposure to ambient ozone in most of California and a few areas in the east/southeast U.S. exceeded the standard of 75 ppb for 7 days (Figure 2, right two panels). The patterns showed by the 90^th ^percentiles were similar to those observed in the 98^th ^percentiles for both PM_2.5 _(Figure 3, left two panels) and ozone (Figure 3, right two panels).

### Population at risk

Table 2 presents the number and percentage of population living in areas where daily potential population exposure to ambient PM_2.5 _and ozone exceeded the standard during 2006 for at least 7, 14 and 28 days by state, with U.S. estimates provided at the bottom. For example, 9% (over 25 million out of 297 million) of the U.S. population lived in areas where daily potential population exposure to ambient PM_2.5 _exceeded the standard and 20% (close to 59 million) lived in areas where daily potential population exposure to ambient ozone exceeded the standard for at least 7 days during 2006, with California having the greatest daily potential population exposures to both ambient PM_2.5 _(11 million, 31% California population) and ozone (13 million, 36% California population). Also shown in Table 2, California was one of a few states which experienced the longest time period of excess exposures (4 weeks during 2006) to both ambient PM_2.5 _(0.77 million) and ozone (6 million). In addition to California, Oregon, Idaho, and Washington also experienced 4 weeks exposure to ambient PM_2.5 _greater than the standard, whereas Texas experienced 4 weeks exposure to ambient ozone exceeding the standard (Table 2).

**Table 2:** The estimated population (percentage) at risk by state in 2006

			PM_2.5 _(%)			Ozone (%)	
			
State	Total Population	7 days	14 days	28 days	7 days	14 days	28 days
**AL**	4598854	670903 (14.6)			1084683 (23.6)		
**AZ**	6166305				706046 (11.5)		
**AR**	2810723				35209 (1.3)		
**CA**	36457524	11160975 (30.6)	4773768 (13.1)	771926 (2.1)	13178688 (36.1)	10814849 (29.7)	6043594 (16.6)
**CO**	4753245				589263 (12.4)		
**CT**	3504809				1032285 (29.5)	58059 (1.7)	
**DE**	853476	119776 (14.0)			682685 (80.0)		
**DC**	581530				581530 (100)	581530 (100)	
**FL**	18089824	20864 (0.1)			286278 (1.6)		
**GA**	9363663	862534 (9.2)			4927293 (52.6)	3671822 (39.2)	
**ID**	1466336	1011626 (69.0)	544057 (37.1)	63198 (4.3)			
**IL**	12831698				87139 (0.7)		
**IN**	6313273						
**IA**	2981803						
**KS**	2763808				10635 (0.4)		
**KY**	4205712						
**LA**	4287605	422831 (9.9)	138256 (3.2)		312766 (7.3)		
**ME**	1321536						
**MD**	5615659	146807 (2.6)			4948656 (88.1)	3242162 (57.7)	
**MA**	6437190				36028 (0.6)		
**MI**	10095452	638666 (6.3)			107717 (1.1)		
**MN**	5166949						
**MS**	2910353				250553 (8.6)		
**MO**	5842473				741140 (12.7)	36948 (0.6)	
**MT**	944423	408598 (43.3)	20334 (2.2)				
**NE**	1767974						
**NV**	2495492				554158 (22.2)	8022 (0.3)	
**NH**	1314886						
**NJ**	8724551	1204022 (13.8)			5720657 (65.6)	254203 (2.9)	
**NM**	1954558						
**NY**	19306071	307218 (1.6)			1261191 (6.5)		
**NC**	8856385				1343735 (15.2)	173227 (2.0)	
**ND**	635721						
**OH**	11477801				259532 (2.3)		
**OK**	3579066				1698046 (47.4)	5781 (0.2)	
**OR**	3700720	2398960 (64.8)	1574925 (42.6)	507823 (13.7)			
**PA**	12440436	2728774 (21.9)	161028 (1.3)		4244008 (34.1)	2610 (0.0)	
**RI**	1067610						
**SC**	4321130				95903 (2.2)		
**SD**	781730						
**TN**	6038551				1220923 (20.2)		
**TX**	23507227				10415151 (44.3)	5132319 (21.8)	414308 (1.8)
**UT**	2549993	162199 (6.4)			328911 (12.9)		
**VT**	623877	4489 (0.7)					
**VA**	7642634				2193047 (28.7)	650405 (8.5)	
**WA**	6395779	1962981 (30.7)	488958 (7.6)	54512 (0.9)			
**WV**	1818265						
**WI**	5556308	1420075 (25.6)			1671 (0.0)		
**WY**	514942	42025 (8.2)	1082 (0.2)				

**U.S**.	297435930	25694323 (8.6)	7702408 (2.6)	1397459 (0.5)	58935527 (19.8)	24631937 (8.3)	6457902 (2.2)

## Discussion

The study has three important results. First, daily BG estimates for ambient PM_2.5 _and ozone concentration were comparable to data observed at monitoring sites, which suggested that inverse-distance weighing was an appropriate method to generate estimates for BGs from gridded data. The second important result was that we generated daily potential population exposure estimates, for both PM_2.5 _and ozone, for various CGUs from BG to state and the U.S. Such population exposure estimates for small areas such as census tracts and counties are very valuable for conducting health impact studies. Moreover, this result highlights the need for investigation and intervention in places with higher estimated daily potential population exposures (not concentrations) and/or longer duration.

The geographical patterns of PM_2.5 _and ozone found (especially at census tract level) were generally consistent with the ranking of most polluted cities (by year round particle pollution and ozone, respectively), provided by the American Lung Association - available at http://www.stateoftheair.org/[33]. The highest daily potential population exposure to ambient PM_2.5 _in the west coast and northwest U.S. may be largely contributed by organic carbon due to high biomass burning such as wildfires, waste burning, and woodstoves [34,35], though nitrate, sulfate, or crustal material may also represent substantial components of PM_2.5 _for the western U.S. [36]. The higher daily potential population exposure to PM_2.5 _in other areas and ozone in general may mainly occur in those megacities or large metropolitan areas where ozone precursors such as volatile organic compounds and oxides of nitrogen produced by heavy traffic (also contribute to organic carbon and nitrite for PM_2.5_) and electric utilities and industrial boilers (also contribute to sulfate and nitrite for PM_2.5_) are concentrated [36,37].

The third important result was that we generated population at risk for each CGU from BG to state and the U.S. based on the NAAQS for PM_2.5 _and ozone. This result provides a hierarchical structure that links hazardous pollution to population affected at different geographic levels. For example, population at risk presented at the state level could be easily traced back to specific CGUs, where information on potential population exposures to ambient air pollutants and population size is needed at smaller CGUs. Such detailed information on potential population exposure level and size of population affected could be used to facilitate communications among public health professionals and/or policy makers across different levels of jurisdiction and help them prioritize resources based on size of population affected and duration of exposures to ambient air pollutants.

There are several limitations. First, we assumed independence among the nearest four grids. This could potentially underestimate the standard errors associated with BG estimates. Second, we used the BG centroid to represent the entire BG area, which on average contains about 39 census blocks [38]. However, in reality, ambient PM_2.5 _or ozone may vary within a BG. Although we thought to convert gridded concentration data to census blocks (the smallest CGU in the U.S.), we were limited to BGs because population data were not available on an annual basis at block level to allow us to generate potential population exposure estimates for larger CGUs. Third, like other studies, we could not account for net population gain or loss for a BG on a daily basis due to population movement across BGs.

An additional limitation was associated with the uncertainty of 36 km- versus 12 km-gridded data. For example, BG estimates from 36 km-grids were slightly more approximate to ground monitoring data than those estimated from 12 km-grids. This may be explained by different sets of input variables included in 36 km- versus 12 km-CMAQ modeling system [18]. We compared 12 km- and 36 km-gridded data against values observed at the nearest monitoring site within specific grids (in an eastern portion of the U.S.) and the comparison statistics (e.g., MAD and R) showed the same pattern as in Table 1 (data not shown): 36 km-gridded data were more approximate to the observed values than 12 km-gridded data. Thus, interpretations of results found must be considered in the context of the limitations of this study.

## Conclusions

We presented a method to allocate gridded data to BGs based on spatial proximities between BGs and their four nearest grids. We used a bottom-up (fine to coarse) strategy to generate CGU population exposures to ambient air pollutants based on BG estimates. Given that BG concentration derived from inverse-distance weighting was comparable to the ground-based monitoring data, using BG as a building block not only provided comparable population exposure estimates across CGUs, but also guaranteed that patterns shown at different geographic levels were consistent, with finer geographic resolution showing more detailed location for potential population exposures to ambient air pollutants. These estimates may be useful in communicating to the general public about the amount and duration of potential population exposures to ambient air pollutants and size of population affected for various geographic levels.

## Abbreviations

BG: census block group; CDC: Centers for Disease Control and Prevention; CGU: census geographic unit; CMAQ: Community Multiscale Air Quality; EPA: U.S. Environmental Protection Agency; EPHTN: Environmental Public Health Tracking Network; MAD: mean absolute deviation; NAAQS: National Ambient Air Quality Standards; PM_2.5_: particulate matter with aerodynamic diameter < 2.5 μm; *R*: correlation coefficient.

## Competing interests

The authors declare that they have no competing interests.

## Authors' contributions

YH was responsible for study design, data analyses, result interpretation and manuscript writing. HF was responsible for study design and result interpretation. MM was responsible for result interpretation and manuscript writing. JQ was responsible for study design, data analyses, and result interpretation. All authors read and approved the final manuscript.

## Supplementary Material

Additional file 1 **Distribution of absolute deviation between BG daily estimates and ground-based monitoring data in an eastern portion of the U.S. (A: PM_2.5 _estimated from 36 km-grid; B: PM_2.5 _estimated from 12 km-grid; C: Ozone estimated from 36 km-grid; D: Ozone estimated from 12 km-grid)**. The file contains distributional statistics of absolute deviation between predicted and observed by season, by year, and by urban/rural status. In addition to mean (MAD), it contains minimum, median, maximum, and the 5^th^, 10^th^, 90^th^, and 95^th ^percentiles of absolute deviation between the two. Click here for file
