# Supplementary material for: U.S. census unit population exposures to ambient air pollutants
Source: Int J Health Geogr. 2012 Jan 12;11:3. doi: 10.1186/1476-072X-11-3 (PMC3274475; doi:10.1186/1476-072X-11-3)
Supplement: Additional file 1 — Distribution of absolute deviation between BG daily estimates and ground-based monitoring data in an eastern portion of the U.S. (A: PM2.5 estimated from 36 km-grid; B: PM2.5 estimated from 12 km-grid; C: Ozone estimated from 36 km-grid; D: Ozone estimated from 12 km-grid). The file contains distributional statistics of absolute deviation between predicted and observed by season, by year, and by urban/rural status. In addition to mean (MAD), it contains minimum, median, maximum, and the 5th, 10th, 90th, and 95th percentiles of absolute deviation between the two. [file 1476-072X-11-3-S1.DOC]

**A: PM_2.5_ estimated from 36km-grid**

|  |  | **M** | **N** | **Min** | **P5** | **P10** | **Median** | **P90** | **P95** | **Max** | **Mean** |
| --- | --- | --- | --- | --- | --- | --- | --- | --- | --- | --- | --- |
| Season | Spring | 1082 | 174892 | 0 | 0.08 | 0.17 | 0.98 | 3.23 | 4.38 | 93.71 | 1.47 |
|  | Summer | 1068 | 173674 | 0 | 0.09 | 0.17 | 1.02 | 3.6 | 4.98 | 78.77 | 1.61 |
|  | Fall | 1077 | 172038 | 0 | 0.08 | 0.17 | 0.94 | 3.09 | 4.22 | 560.78 | 1.43 |
|  | Winter | 1098 | 171491 | 0 | 0.09 | 0.18 | 1.06 | 3.71 | 5.2 | 108.98 | 1.67 |
| Year | 2001 | 813 | 113401 | 0 | 0.09 | 0.18 | 1.06 | 3.71 | 5.24 | 560.78 | 1.69 |
|  | 2002 | 899 | 127779 | 0 | 0.09 | 0.17 | 0.99 | 3.38 | 4.67 | 78.77 | 1.55 |
|  | 2003 | 894 | 117879 | 0 | 0.09 | 0.17 | 1.01 | 3.44 | 4.71 | 108.98 | 1.56 |
|  | 2004 | 833 | 116326 | 0 | 0.08 | 0.17 | 0.97 | 3.25 | 4.43 | 129.51 | 1.47 |
|  | 2005 | 860 | 111552 | 0 | 0.09 | 0.18 | 1.04 | 3.49 | 4.8 | 66.63 | 1.58 |
|  | 2006 | 807 | 105158 | 0 | 0.08 | 0.16 | 0.92 | 3.15 | 4.36 | 68.1 | 1.42 |
| Urban/Rural | Urban | 448 | 288773 | 0 | 0.08 | 0.17 | 1 | 3.55 | 4.94 | 108.98 | 1.6 |
|  | Suburban | 448 | 302462 | 0 | 0.08 | 0.17 | 0.98 | 3.26 | 4.46 | 93.71 | 1.48 |
|  | Rural | 209 | 100114 | 0 | 0.09 | 0.19 | 1.05 | 3.45 | 4.7 | 560.78 | 1.58 |
|  | Unknown | 2 | 746 | 0.01 | 0.16 | 0.28 | 1.28 | 3.74 | 5.17 | 13.06 | 1.77 |

**B: PM_2.5_ estimated from 12km-grid**

|  |  | **M** | **N** | **Min** | **P5** | **P10** | **Median** | **P90** | **P95** | **Max** | **Mean** |
| --- | --- | --- | --- | --- | --- | --- | --- | --- | --- | --- | --- |
| Season | Spring | 1082 | 174892 | 0 | 0.14 | 0.28 | 1.57 | 4.93 | 6.53 | 125.23 | 2.24 |
|  | Summer | 1068 | 173674 | 0 | 0.14 | 0.28 | 1.64 | 5.57 | 7.47 | 93.58 | 2.48 |
|  | Fall | 1077 | 172038 | 0 | 0.13 | 0.26 | 1.47 | 4.66 | 6.29 | 604.69 | 2.14 |
|  | Winter | 1098 | 171491 | 0 | 0.14 | 0.27 | 1.56 | 5.09 | 6.93 | 109.21 | 2.31 |
| Year | 2001 | 813 | 113401 | 0 | 0.12 | 0.25 | 1.47 | 5 | 6.75 | 604.69 | 2.23 |
|  | 2002 | 899 | 127779 | 0 | 0.14 | 0.27 | 1.56 | 5.04 | 6.78 | 92.51 | 2.31 |
|  | 2003 | 894 | 117879 | 0 | 0.14 | 0.28 | 1.61 | 5.22 | 7.05 | 109.21 | 2.36 |
|  | 2004 | 833 | 116326 | 0 | 0.14 | 0.28 | 1.57 | 4.94 | 6.59 | 150.4 | 2.25 |
|  | 2005 | 860 | 111552 | 0 | 0.15 | 0.29 | 1.65 | 5.33 | 7.19 | 93.58 | 2.41 |
|  | 2006 | 807 | 105158 | 0 | 0.12 | 0.25 | 1.48 | 4.84 | 6.54 | 81 | 2.18 |
| Urban/Rural | Urban | 448 | 288773 | 0 | 0.13 | 0.26 | 1.51 | 4.97 | 6.74 | 109.21 | 2.25 |
|  | Suburban | 448 | 302462 | 0 | 0.14 | 0.27 | 1.56 | 4.95 | 6.61 | 125.23 | 2.25 |
|  | Rural | 209 | 100114 | 0 | 0.14 | 0.29 | 1.72 | 5.65 | 7.57 | 604.69 | 2.55 |
|  | Unknown | 2 | 746 | 0 | 0.12 | 0.28 | 1.61 | 5.39 | 7.13 | 57.72 | 2.42 |

**C: Ozone estimated from 36km-grid**

|  |  | **M** | **N** | **Min** | **P5** | **P10** | **Median** | **P90** | **P95** | **Max** | **Mean** |
| --- | --- | --- | --- | --- | --- | --- | --- | --- | --- | --- | --- |
| Season | Spring | 1038 | 375224 | 0 | 0.19 | 0.38 | 2.12 | 6.16 | 7.9 | 68.88 | 2.85 |
|  | Summer | 1039 | 452609 | 0 | 0.24 | 0.48 | 2.68 | 7.65 | 9.81 | 61.47 | 3.55 |
|  | Fall | 1040 | 335336 | 0 | 0.2 | 0.39 | 2.19 | 6.33 | 8.19 | 56.8 | 2.93 |
|  | Winter | 457 | 171915 | 0 | 0.19 | 0.38 | 2.11 | 5.94 | 7.59 | 40.81 | 2.76 |
| Year | 2001 | 790 | 195035 | 0 | 0.21 | 0.43 | 2.4 | 6.98 | 9 | 56.8 | 3.22 |
|  | 2002 | 856 | 223175 | 0 | 0.21 | 0.43 | 2.4 | 6.95 | 9.01 | 58.31 | 3.23 |
|  | 2003 | 873 | 226947 | 0 | 0.21 | 0.42 | 2.32 | 6.63 | 8.46 | 61.47 | 3.07 |
|  | 2004 | 896 | 232081 | 0 | 0.2 | 0.4 | 2.23 | 6.45 | 8.33 | 47.19 | 2.98 |
|  | 2005 | 883 | 230270 | 0 | 0.2 | 0.41 | 2.28 | 6.71 | 8.68 | 56.09 | 3.09 |
|  | 2006 | 887 | 227576 | 0 | 0.2 | 0.4 | 2.24 | 6.55 | 8.45 | 68.88 | 3.01 |
| Urban/Rural | Urban | 176 | 216682 | 0 | 0.22 | 0.45 | 2.54 | 7.39 | 9.51 | 61.01 | 3.41 |
|  | Suburban | 410 | 555995 | 0 | 0.21 | 0.41 | 2.29 | 6.6 | 8.52 | 61.47 | 3.06 |
|  | Rural | 463 | 555923 | 0 | 0.2 | 0.4 | 2.24 | 6.54 | 8.42 | 68.88 | 3.01 |
|  | Unknown | 6 | 6484 | 0 | 0.17 | 0.36 | 1.99 | 5.73 | 7.24 | 23.24 | 2.65 |

**D: Ozone estimated from 12km-grid**

|  |  | **M** | **N** | **Min** | **P5** | **P10** | **Median** | **P90** | **P95** | **Max** | **Mean** |
| --- | --- | --- | --- | --- | --- | --- | --- | --- | --- | --- | --- |
| Season | Spring | 1038 | 375224 | 0 | 0.24 | 0.49 | 2.73 | 7.55 | 9.53 | 66.32 | 3.55 |
|  | Summer | 1039 | 452609 | 0 | 0.3 | 0.6 | 3.29 | 9.1 | 11.62 | 63.62 | 4.29 |
|  | Fall | 1040 | 335336 | 0 | 0.25 | 0.5 | 2.78 | 7.75 | 9.87 | 58.96 | 3.64 |
|  | Winter | 457 | 171915 | 0 | 0.24 | 0.49 | 2.65 | 7.15 | 9.04 | 39.13 | 3.4 |
| Year | 2001 | 790 | 195035 | 0 | 0.31 | 0.63 | 3.48 | 9.62 | 12.23 | 63.62 | 4.53 |
|  | 2002 | 856 | 223175 | 0 | 0.26 | 0.52 | 2.9 | 8.14 | 10.47 | 58.21 | 3.83 |
|  | 2003 | 873 | 226947 | 0 | 0.26 | 0.52 | 2.86 | 7.76 | 9.84 | 62.63 | 3.69 |
|  | 2004 | 896 | 232081 | 0 | 0.25 | 0.49 | 2.71 | 7.42 | 9.4 | 49.49 | 3.51 |
|  | 2005 | 883 | 230270 | 0 | 0.26 | 0.52 | 2.88 | 7.97 | 10.13 | 52.86 | 3.75 |
|  | 2006 | 887 | 227576 | 0 | 0.25 | 0.5 | 2.79 | 7.74 | 9.84 | 66.32 | 3.63 |
| Urban/Rural | Urban | 176 | 216682 | 0 | 0.27 | 0.54 | 2.94 | 8.01 | 10.18 | 63.62 | 3.8 |
|  | Suburban | 410 | 555995 | 0 | 0.25 | 0.5 | 2.76 | 7.56 | 9.56 | 58.21 | 3.57 |
|  | Rural | 463 | 555923 | 0 | 0.27 | 0.55 | 3.05 | 8.64 | 11.1 | 66.32 | 4.05 |
|  | Unknown | 6 | 6484 | 0 | 0.23 | 0.49 | 2.75 | 7.93 | 10.28 | 38.67 | 3.67 |

M: The number of monitoring sites; N: The number of records; Min, P5, p10, p90, p95, and Max: The minimum, 5^th^, 10^th^, 90^th^, 95^th^ percentiles, and maximum of absolute deviation; Spring: March-May; Summer: June-August; Fall: September-November; and Winter: December-February.
